# Short Course Hypofractionated Radiotherapy for Frail or Elderly Patients With Meningioma

**DOI:** 10.7759/cureus.8604

**Published:** 2020-06-13

**Authors:** Nasim Sarhan, Lulwah Abduljabbar, Normand Laperriere, David Shultz, Mohammed Asha, Gelareh Zadeh, Barbara-Ann Millar, Derek S Tsang

**Affiliations:** 1 Radiation Oncology, Princess Margaret Cancer Center, Toronto, CAN; 2 Radiation Oncology, King Fahad Specialist Hospital, Dammam, SAU; 3 Neurosurgery, Toronto Western Hospital, Toronto, CAN

**Keywords:** short course, hypofractionated radiotherapy, frail, elderly, meningioma, radiation, radiation fractionation

## Abstract

Purpose/Objective(s)

The incidence of intracranial meningiomas increases with age. The standard of care treatment is complete surgical excision, followed by radiation therapy (RT) if indicated. However, six weeks of RT can be challenging for elderly or frail patients. The purpose of this study was to determine if short course RT is safe and effective in elderly patients with meningioma.

Materials/Methods

We performed a retrospective analysis of patients with meningioma treated with short course beam RT (5-15 fractions) at a single institution. Seventeen patients (94%) received 4005 cGy over 15 fractions and one patient (6%) received 2500 cGy over five fractions. Study endpoints were treatment toxicity (edema), progression-free (PFS) and overall survival (OS).

Results

Eighteen patients with histologically proven (n = 12) or radiologically presumed meningioma (n = 6, presumed grade I) were identified. Median age at treatment was 85 years (66-95 years). There were eight, eight and two patients with grade I, II and III tumours, respectively. Eight patients (44%) had radiologic edema prior to RT. Six (33%) required dexamethasone treatment during RT and the dose was increased during RT for two patients. Fourteen patients had reduced or no edema post-RT and 13 patients had stable or improving symptoms post-RT. Six patients had disease progression (five in-field, one out-of-field). Median PFS was 3.3 and 0.9 years for grade I and II/III tumours, respectively (p = 0.014). Median OS was 3.3 and 2.5 years for grade I and II/III tumours, respectively (p = 0.12).

Conclusion

Short course RT for elderly patients with meningioma is well-tolerated and can offer disease control for some patients, particularly those with grade I tumours.

## Introduction

Intracranial meningiomas originate from the meninges and are classified into three grades according to the World Health Organization (I, II or III) [1]. Grade I tumors are slow-growing and are often found incidentally by CT or MRI [2,3]. The incidence of meningiomas increases with age, with the highest incidence in individuals age 80 or older [4,5].

The standard of care treatment for meningiomas is complete surgical excision, followed by radiation therapy (RT), which is indicated in cases of higher-grade histology, subtotal resection, and/or recurrence [6,7]. For unresectable lesions or lesions with high expected morbidity with a surgical approach, definitive RT is the standard treatment [8]. However, neurosurgical intervention or fractionated radiation treatment (up to six weeks) can be challenging for elderly or frail patients. A systematic review of hypofractionated radiotherapy (RT) revealed high local control of 90% or higher, as well as a low probability of late toxicities (10%) [9]. However, there is little reported data regarding hypofractionated RT in elderly or frail patients with meningioma.

The primary objective of this study was to determine if hypofractionated external beam RT treatment is well tolerated in elderly patients with meningioma and to report early clinical outcomes of this treatment.

## Materials and methods

This was a retrospective, single-institution cohort study of patients with a histologically proven or radiologically diagnosed intracranial meningioma treated with short course, hypofractionated external beam radiotherapy (5-15 fractions) at the Princess Margret Cancer Centre in Toronto, Canada from 2006 to 2019. Patients who received conventionally fractionated radiotherapy (1.8-2 Gy) or stereotactic radiosurgery (SRS) for meningioma were excluded. Eligible patients were identified using the clinical radiotherapy record-and-verify system. The study was approved by the research ethics board of the University Health Network.

Treatments

Elderly or frail patients who were deemed intolerant of conventionally fractionated RT over six weeks and also unsuitable for SRS (large tumour size >3 cm or atypical/anaplastic histology) were offered hypofractionated RT. Photon external beam therapy was used for all patients. Patients were simulated using CT with MR registration. The gross tumour volume included all viable tumour and tumour bed (if post-operative). A clinical target volume of 0-5 mm was applied (0 mm if no surgery, 5 mm if post-operative). A planning target volume of 3 mm was used with daily cone-beam CT image guidance, or 5 mm without. RT was delivered using inverse-planned intensity modulated radiotherapy or volumetric modulated arc therapy. Almost all patients received 40 Gy in 15 fractions (one patient received 25 Gy in five fractions).

Analysis

The study database was closed on August 1, 2019 for analysis. Study endpoints were treatment toxicity (radiologic or symptomatic edema), progression-free survival (PFS) and overall survival (OS). Baseline characteristics and frequencies were reported descriptively. Comparison of continuous values was done using the Wilcoxon rank sum test. Median follow-up was determined by reversing the censoring variable. The Kaplan-Meier method was used to estimate PFS and OS from the first day of hypofractionated RT. Survival comparisons were made using the log-rank test. Cumulative incidence of local failure (LF) was estimated, after stratifying by tumor grade; deaths without known disease progression were treated as a competing event. Comparisons of LF were done using Gray’s test. Living patients or those lost-to-follow-up were censored. Statistical analyses were performed using SAS version 9.4 (Cary, NC, USA). Calculations for equivalent dose in 2-Gy fractions (EQD_2_) were done using the linear-quadratic equation with the RBApp tool [10].

## Results

Eighteen patients were included in the analysis (Table 1); there were nine females and nine males. All patients received hypofractionated, short course radiotherapy (RT), as part of definitive treatment of histologically proven meningioma (n = 12) or radiologically presumed meningioma (n = 6). The median age at the time of RT was 85 years (range 66-95). Twelve had previous surgery while four had previous radiotherapy, all for primary intracranial meningioma. Most individuals received 40 Gy in 15 fractions; one patient received 25 Gy in five fractions due to advanced age (95 years), as this individual desired a shorter treatment regimen.

**Table 1:** Elderly patients treated with hypofractionated RT for meningioma Y: yes; N: no; NA: not available; IT: infratentorial; R: reduced; S: stable; IN: infield; OUT: outfield; O: observation; P: palliative care; SU: surgery; RT: radiotherapy; DOD: dead of disease.

Patient number	Age at RT	Prior Surgery	Prior RT	WHO grade	Location		Volume	Dose	Edema		Dexamethasone		Symptomatic worsening post-RT	Disease progression			Salvage treatment	Follow-up time	Vital status
	(y)				IT	Parafalcine	(cc)	(Gy)	Pre-RT	Post-RT	During RT	Increased during RT		Present	Location	Time from RT			
1	76	Y	N	II	N	N	36	40	Y	N	N		N	Y	IN	0.2	O	1.6	DOD
2	89	Y	N	II	N	N	117	40	Y	NA	Y	N	N	N				0.05	Dead
3	92	N	N	I	N	Y	56	40	Y	R	Y	Y	N	Y	IN	2.6	O	2.6	Dead
4	89	Y	N	III	N	N	227	40	Y	N	N		N	Y	IN	1.7	P	2.5	DOD
5	85	Y	N	I	N	N	38	40	Y	S	N		Y	Y	IN	0.6	SU	2.8	Dead
6	89	N	Y	I	N	N	2	40	N	N	N		N	N				3.9	Alive
7	66	Y	N	II	N	N	4	40	N	N	N		N	Y	OUT	0.3	RT	3.8	Alive
8	88	N	N	I	N	N	9	40	N	N	N		N	N				3.3	Dead
9	81	N	Y	I	N	N	55	40	N	N	N		N	N				3.5	Alive
10	81	Y	N	II	N	Y	93	40	Y	NA	Y	N	NA	NA				0.2	Dead
11	92	N	N	I	N	Y	29	40	N	N	N		N	N				0.6	Alive
12	79	Y	N	II	N	Y	15	40	Y	N	N		Y	Y	IN	1.5	SU	2.5	Alive
13	72	Y	N	II	N	Y	31	40	N	N	N		N	N				1.9	Alive
14	79	Y	N	II	Y	N	20	40	N	N	N		N	N				1.4	Alive
15	85	Y	N	III	N	Y	165	40	N	NA	Y	N	NA	N				0.2	Dead
16	85	Y	N	I	N	N	27	40	N	N	N		N	N				1	Alive
17	84	N	N	I	N	N	16	40	N	N	Y	Y	N	N				0.8	Alive
18	95	Y	Y	II	N	N	21	25	Y	Y	Y	N	Y	N				0.3	Alive

Eight patients (44%) had radiological edema prior to radiotherapy. A total of six patients required dexamethasone treatment before, during or after RT. In two patients, the dose of dexamethasone was increased during RT to control neurologic symptoms of edema. Fourteen patients had reduced or no edema post RT. Thirteen patients had stable or improving symptoms post RT. Median disease volume was 75 cc and 28 cc in those who did or did not require dexamethasone at any time (p = 0.14). There was no clear association between parafalcine location and edema post RT.

Outcomes

Six patients had disease progression (five in-field, and one out of-field recurrence). The crude rate of disease progression was 33%. Upon progression, two patients were observed, two patients had surgery, one was referred to palliative care, and one who had out of field progression received another course of RT; this individual (patient 7) remains alive without evidence of disease, 3.7 years after the first course of RT. All patients completed the prescribed course of irradiation; however, patient 2 died after the last fraction of RT in the context of a lower gastrointestinal bleed with concomitant thrombocytopenia and suspected immune thrombocytopenia.

**Figure 1:**
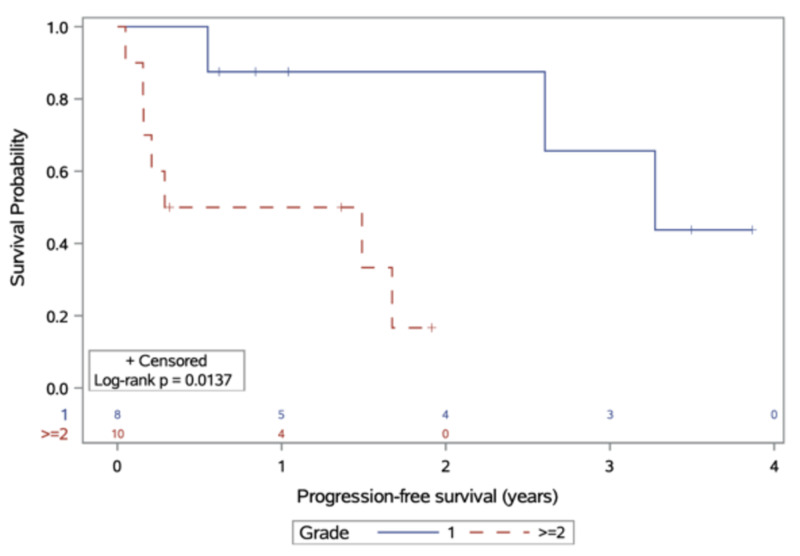
Progression-free survival, stratified by meningioma grade at diagnosis

Median follow-up was 3.5 years. Median time-to-progression after RT was 3.3 and 0.9 years for those with grade I and II/III tumours, respectively (p = 0.014; Figure 1). One-year PFS was 88% and 50% for grade I and II/III tumours, respectively.

**Figure 2:**
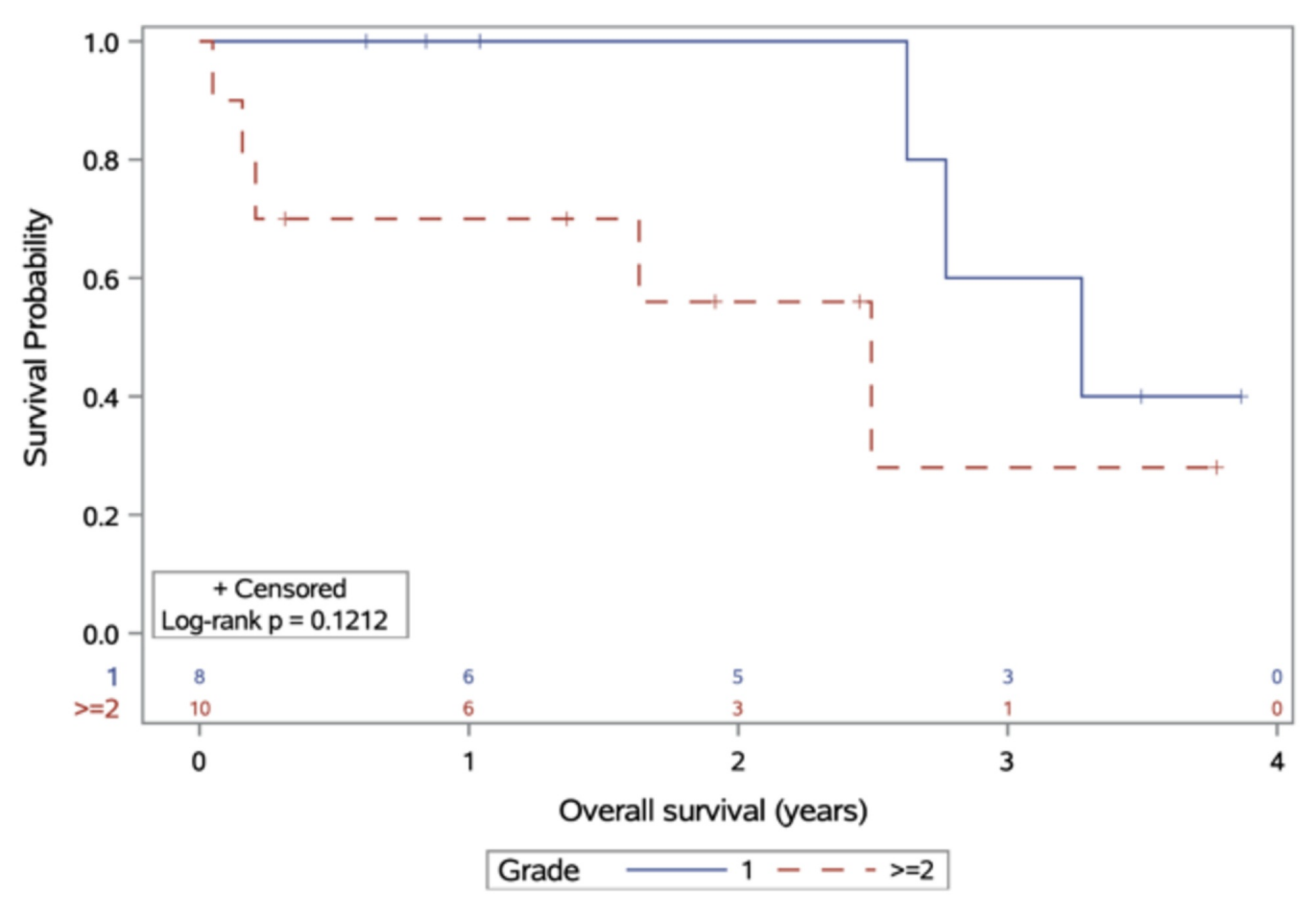
Overall survival, stratified by meningioma grade at diagnosis

At the time of database closure, 10 patients were alive; none were lost to follow-up. Eight were living without disease while two remained alive with disease progression after RT. Median survival for patients with meningioma grade I and II/III after RT was 3.3 and 2.5 years, respectively (p = 0.12), with one-year OS of 100% and 70% for grade I and II/III tumours (Figure 2). Three-year OS was 60% and 28% for grade I/II tumours, respectively. Two patients died due to meningioma; one patient died of stroke. Cause of death was not available for five remaining patients. All deceased patients except one patient had disease volume of at least 36 cc. Both patients with anaplastic (grade III) meningioma had died.

**Figure 3:**
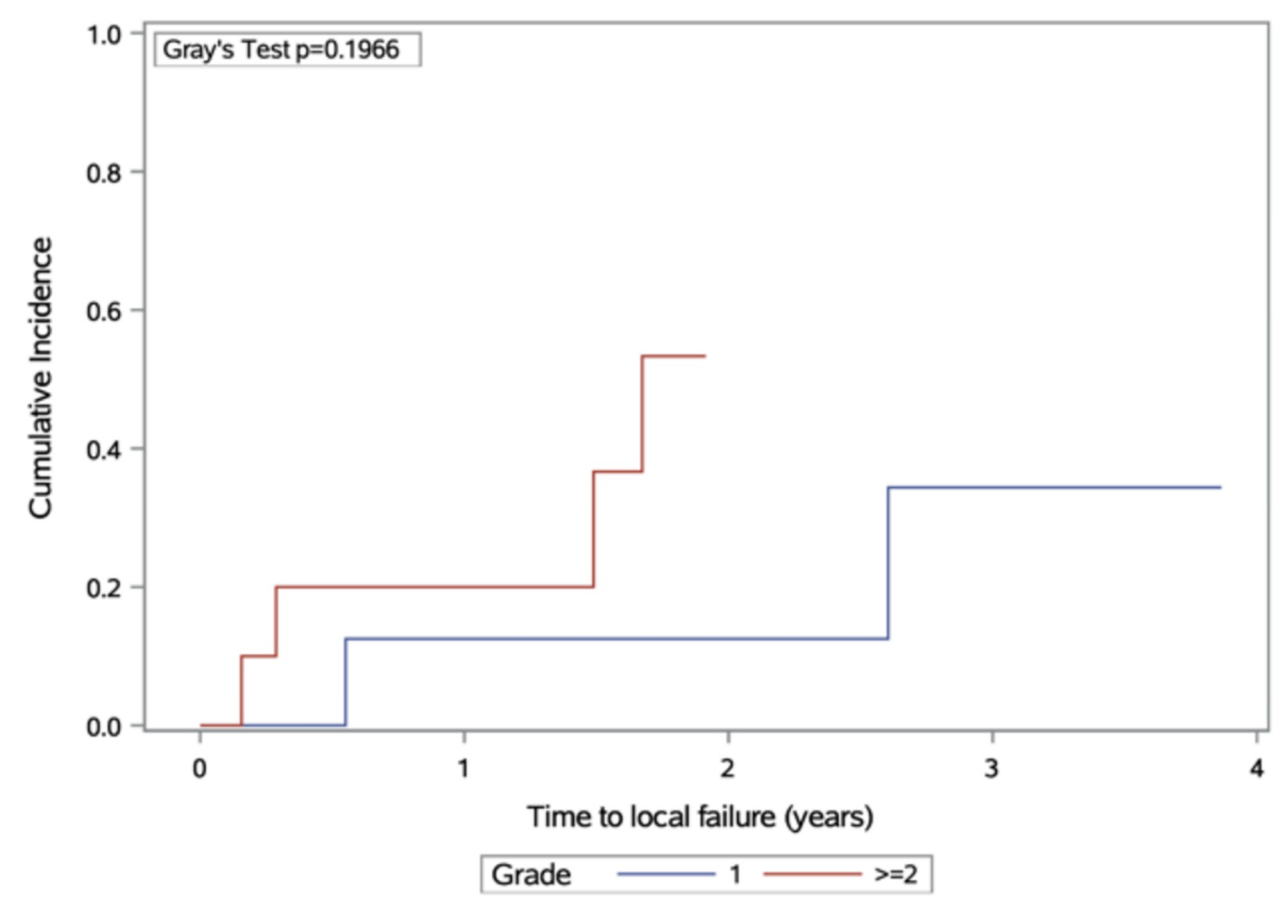
Cumulative incidence of local failure, stratified by meningioma grade at diagnosis

Estimates of local failure are presented in Figure 3. There was a higher cumulative incidence of local failure with grade II/III tumours, but this did not achieve statistical significance (p = 0.20).

## Discussion

This study evaluated the outcomes of frail or elderly patients treated with hypofractionated, short-course RT for histologically proven or radiologically presumed meningioma. Patients with grade II/III experienced poor PFS and OS, while those who had RT for grade I meningioma experienced longer PFS. Individuals with grade II/III histology may have a higher incidence of local failure, but this did not achieve statistical significance due to our small sample size. Nonetheless, we believe that hypofractionated RT had temporary, palliative benefit for those with grade II/III tumours in this challenging-to-treat patient subgroup. The data from this study are valuable because they demonstrate the efficacy of short course, hypofractionated RT radiotherapy in preventing symptom progression and achieving disease control in a majority of elderly patients, particularly those with grade I tumours.

There are limited prior reports on the use of hypofractionated RT for meningioma, though none have focused exclusively on the elderly subgroup and most had evaluated 5-fraction regimens. Nguyen et al. performed a systematic review of hypofractionated stereotactic radiotherapy and found low rates of late toxicity and high grades of crude local control [9]. Radiologic response was reported; 96% of tumors treated remained stable or decreased in size, while an increase in size occurred in 4%. However, duration of follow-up, like the present study, was short. Chung et al. reviewed and compared stereotactic radiosurgery (SRS) versus fractionated stereotactic radiotherapy (FSRT) for grade I meningioma; they found that both treatments were comparable, with a correlation between tumor volume and probability of complications [11]. There was a lack of consensus regarding the best dose-fractionation regimen for meningioma, as there have been no prospective randomized trials comparing different dose fractionations. Thus, we have based decisions about RT dose-fractionation on tumor and patient factors that include: age and performance status, previous irradiation, comorbidities, tumor volume, and intent of treatment.

The hypofractionated regimens used for the patients in this study provide a slightly lower or similar biologically effective dose as compared to conventional dose schedules, such as 50 Gy in 25 fractions or 54 Gy in 30 fractions. A comparison of the equivalent dose in 2 Gy fractions (EQD_2_) is shown in Table 2. Assuming an alpha/beta (α/β) ratio of 2 or 3 for a slow growing, indolent tumor such as meningioma, the abbreviated regimens appear to have slightly lower or similar EQD_2_.

**Table 2:** Comparison of various dose-fractionation schedules for meningioma EQD_2_ equivalent dose in 2 Gy fractions

Fractionation schedule	EQD_2_ (α/β = 2)	EQD_2_ (α/β = 3)
50 Gy in 25 fractions	50 Gy	50 Gy
54 Gy in 30 fractions	51.3 Gy	51.8 Gy
40 Gy in 15 fractions	46.7 Gy	45.3 Gy
25 Gy in 5 fractions	43.8 Gy	40 Gy

To our knowledge, this is the first published study of short course, hypofractionated RT focused on elderly patients with meningioma. In elderly patients with glioblastoma, 40 Gy in 15 fractions was non-inferior to 60 Gy in 30 fractions [12]. A summary of selected studies on hypofractionated RT for meningioma is provided in Table 3. Haghighi et al. demonstrated that hypofractionated radiotherapy was safe, with low rates (3%) of cranial nerve (CN) deficit, and the possibility that RT could improve pre-existing CN deficit in patients with meningioma [13]. Maranzano et al., in an update of previous work published by Trippa et al. [14], suggested that moderately hypofractionated radiotherapy was a reasonable treatment in patients with recurrent and inoperable meningioma [15]; moreover, their finding of poorer outcomes with atypical meningiomas is consistent with our present work.

**Table 3:** Selected published studies on hypofractionated radiotherapy for meningioma *Mean values

Study author, year	Age (median)	Number of patients	RT dose (Gy/fractions)	Volume (median)	Stable or improved symptoms	PFS (% or median)
Haghighi et al., 2015 [13]	52*	57 (meningioma only)	37.5-40/15	7 cc	95%	5 years 98% 10 years 93%
Maranzano et al., 2015 [15]	65	77	45/15 42/14	23 cc	97%	5 years 84% 10 years 84%
Gorman et al., 2008 [16]	56	38	35-40/15	8 cc	87%	100%
Kaul et al., 2014 [17]	59	92 (FSRT only)	33.2-42 Gy, 2.2-5 Gy/fraction	15 cc*	NA	3 years 92.4% 5 years 80.9%
Present study, 2020	85	18	40/15 25/5	30 cc	81%	3.3 y (grade I) 2.5 y (grade II/III)

Our cohort included patients with larger tumour volumes (median 30 cc) in comparison with the other studies listed in Table 2. The cohorts by Haghighi et al. [13] and Gorman et al. [16] included very small median tumor volumes and had the highest PFS; on the other hand, Maranzano et al. [15] included bigger tumor volumes and had lower PFS, which is consistent with our results. The relation between tumor volume and symptom control demonstrated an inverse relationship in all studies except one (Maranzano et al. [15]). Thus, hypofractionated RT may be best suited to patients with small tumors, as this may optimize disease control while keeping the risk of toxicity low. Although our study is limited by a small sample size and few patients with long-term follow-up, our data nonetheless demonstrate early safety and feasibility of hypofractionated RT in very elderly patients, as the median age of patients in our study is at 20-30 years older than the median ages in other studies. Future work should aim to better determine clinical factors that make patients most suited to hypofractionated RT (as compared to surgery or conventionally fractionated RT), as well as inclusion of data from multiple institutions to increase generalizability of study findings.

## Conclusions

In conclusion, we demonstrate that hypofractionated RT is a feasible treatment option for elderly patients with meningioma to minimize the burden of repeated treatment visits. This treatment was well tolerated and can offer disease control for many patients, particularly those with grade I tumors.
